# Damage Detection Based on Static Strain Responses Using FBG in a Wind Turbine Blade

**DOI:** 10.3390/s150819992

**Published:** 2015-08-14

**Authors:** Shaohua Tian, Zhibo Yang, Xuefeng Chen, Yong Xie

**Affiliations:** 1 The State Key Laboratory for Manufacturing Systems Engineering, Xi’an 710049, China; E-Mails: shaua@126.com (S.T.); chenxf@mail.xjtu.edu.cn (X.C.); 2 School of Mechanical Engineering, Xi’an Jiaotong University, Xi’an 710049, China; 3 State Key Laboratory of Strength and Vibration for Mechanical Structures, Xi’an Jiaotong University, Xi’an 710049, China; E-Mail: yxie@mail.xjtu.edu.cn; 4 School of Aerospace, Xi’an Jiaotong University, Xi’an 710049, China

**Keywords:** wind turbine blade, CSD, FIF, SEM, MDDE

## Abstract

The damage detection of a wind turbine blade enables better operation of the turbines, and provides an early alert to the destroyed events of the blade in order to avoid catastrophic losses. A new non-baseline damage detection method based on the Fiber Bragg grating (FBG) in a wind turbine blade is developed in this paper. Firstly, the Chi-square distribution is proven to be an effective damage-sensitive feature which is adopted as the individual information source for the local decision. In order to obtain the global and optimal decision for the damage detection, the feature information fusion (FIF) method is proposed to fuse and optimize information in above individual information sources, and the damage is detected accurately through of the global decision. Then a 13.2 m wind turbine blade with the distributed strain sensor system is adopted to describe the feasibility of the proposed method, and the strain energy method (SEM) is used to describe the advantage of the proposed method. Finally results show that the proposed method can deliver encouraging results of the damage detection in the wind turbine blade.

## 1. Introduction

Structural health monitoring (SHM) is expected to play a major role in the development of large wind turbines with higher efficiency and lower cost-of-energy [1]. Li *et al.* [2] gave a comprehensive review on the damage detection methods for wind turbine blades. Taylor *et al.* [3] presented an ongoing work to implement real-time SHM systems for operational research-scale wind turbine blades with piezoelectric active sensors. Park [4] designed a real-time monitoring system with FBG sensors for a 2 MW wind turbine (type U88). Those wind turbines are usually subjected to severe operational loads, therefore they requires stringent safety measures and more frequent maintenance, but it is quite difficult and costly to perform inspection and maintenance work on these large wind turbines, mainly because of their height and limitations associated with the remoteness of the installation locations, such as offshore wind farms. Clearly, obtaining precise and real time information using SHM systems can be invaluable for improving safety, for lowering the frequency of sudden breakdowns and, more importantly, for moving away from a costly schedule-based maintenance toward a cost-effective and condition-based maintenance; all factors lead to the significant reduction in the operational cost of wind turbines [5].

A precondition for the wind turbine SHM is the understanding of loads, which is important to manufacturers and wind-farm owners. For the manufacturer, a better understanding of the loads enables improved designs. For the wind-farm operator, understanding loads caused by the damage enables the better detection of potentially damaging situations, and it provides an early alert to “blade throw” events, which could have catastrophic consequences for anything in the surrounding area.

The Fiber Bragg grating (FBG) shows an advantage over the conventional strain sensor in understanding loads of the wind turbine blade. Typically wind turbines are designed for a 20-year operational life, and during that time the sensor will undergo approximately 60 million cycles. A conventional strain sensor will typically fail after less than 60,000 cycles, but the FBG shows no degradation in the performance after 100 million cycles. Therefore, once embedded within the wind turbine blade during the manufacturing process, the FBG is there for the life of the wind turbines, with no need for servicing or recalibration [6].

The development of the wind turbine SHM using FBG strain sensors has received wide attention recently due to the increasing interest in renewable energy. Kim *et al*. [7] employed the operational modal analysis to construct a displacement-strain transformation matrix for a real-time shape estimation technique, and the developed technique is applied to a wind turbine blade, in which the FBG sensors are embedded. Arsenault *et al.* [8] calculated the power spectra density based on the strain response of the wind turbine blade, a lumped mass was attached near the tip of the blade to simulate the damage, and the comparison between the lumped mass-added blade power spectra and that of the intact blade was made in order to detect damage. Nichols *et al.* [9] detected the presence of damage-induced nonlinearities in composite structures using only the structural vibration response, and the damage was assumed to change the coupling between different locations on the structure from linear to nonlinear; two informational metrics, the time-delayed mutual information and time-delayed transfer entropy, were obtained from the obtained time-series data, so the presence of the impact damage was detected in a thick composite sandwich plate. Lau *et al.* [10] employed the FBG as a structural health monitoring device for fiber reinforced plastic materials by either embedding into, or bonding onto, the structures, and the accuracy of the strain with the FBG sensor is highly dependent on the bonding characteristics among the bare optical fiber, protective coating, adhesive layer and host material. Okabe *et al.* [11] developed small-diameter FBG sensors for embedding the laminated composite plate without deterioration of the mechanical properties. Tsuda [12] constructed two types of FBG ultrasonic sensing for the damage detection in carbon-fiber reinforced plastics. Therefore, it can be concluded that the key procedure of the SHM is to extract the damage-sensitive feature from the measured parameter and track those features with the presence of damage, with the primary goal of the SHM to combine advances in both sensing and data analysis in order to produce an automated system capable of detecting damage without requiring visual inspecting [9].

The presence of damage is always accompanied with the local variation on strain parameters in the vicinity, but those in the intact region remain unchanged, so the damage sensitive feature must be able to measure and display this dissimilarity of the strain parameter due to damage. As a nonparametric test statistic method, the Chi-square distribution (CSD) can provide a probabilistic procedure for testing the hypothesis that two probabilistic distributions have been generated from the same underlying distribution, and then the structural dissimilarity is measured based on the empirical estimates. Rubner *et al.* [13] employed the CSD to complete the texture dissimilarity measure. Mathiassen and Skavhaung [14] employed it to measure the texture dissimilarity in composites, and then Puzicha [15] utilized it to evaluate the dissimilarity for the color and texture. In this study, the CSD is adopted to be as an effective damage-sensitive feature and individual information source for the damage detection.

To fuse and optimize explicit information of the individual information source CSD on the damage location, an information fusion method addressed as a feature information fusion (FIF) method is developed, and it can combine data from multiple information sources and related information from associated databases, in order to achieve improved accuracies and more specific inferences than could be achieved by the use of a single source alone. Each source makes its local decision based on its observation, and the local decision of each source is sent to the fusion center, where the global decision based on the local decisions is obtained.

In this paper, a damage detection method in the wind turbine blade based on the FBG is presented. Firstly, the strain response under varying levels of static load is measured using a distributed sensor network, consisting of twelve FBG sensors adhered to the windward side of the wind turbine blade, which is consistent with the situation in service. The presence of damage increases the dissimilarity among strain responses under different levels of static loads, so the dissimilarity is measured by the damage-sensitive feature CSD, which is adopted as the individual information source, and then the local decision on the damage detection is made. Secondly, the feature information fusion (FIF) method is proposed to fuse and optimize information in the above individual information source in order to obtain the global and optimal decision for the damage detection, so the damage is detected accurately on the basis of the global decision. Furthermore, the mean damage detection error (MDDE) is used to describe the damage detection error quantitatively, and the strain energy method (SEM) is employed to show the advantage of the proposed method. Finally, the proposed method does not require the full strain history to detect the current damage, unlike the FBG peak wavelength measurement, and results show that it can deliver encouraging results of the damage detection in the wind turbine blade.

## 2. Damage Detection Method

In this section, a damage detection method based on the CSD and FIF is developed. The first procedure is to adopt the CSD as the damage-sensitive feature and individual information source, and the next is to employ the FIF to obtain a global and optimal decision for the damage detection.

### 2.1. The Chi-Square Distribution

The Chi-square distribution is a widely used tool in the statistics and pattern recognition to measure the texture dissimilarity, and it is defined as follows:

(1) $$CSD\left(p,q\right)=\sum_{j=1}^{N}\sum_{l=0}^{L-1}\frac{{\left[q_{j}\left(l\right)-p_{j}\left(l\right)\right]}^{2}}{q_{j}\left(l\right)+p_{j}\left(l\right)}$$

where p(l) and q(l) represent probability density functions respectively, L represents the element number, *j* denotes the feature dimension, and *N* is the number of the feature dimension.

On the right side of Equation (1), the numerator represents the difference between two probability density function, and the denominator represents the sum of them, so it can be concluded that the CSD indicates the difference between two probability density functions and it can be used to measure the dissimilarity among them. The presence of damage is always accompanied with the variation on local strain parameters, and corresponding ones in the intact region remain unchanged, so the CSD can be used to indicate the difference among strain responses with the presence of damage and be regarded as an effective damage-sensitive feature.

When the CSD is used to be an damage-sensitive feature of the wind turbine blade, the strain responses under the static load are measured using the distributed strain sensor system, and then the normalization of the strain response is conducted; therefore, the strain response can be regarded as two probability distribution functions, suppose that $\varepsilon^{l}\left(x\right)$ and $\varepsilon^{m}\left(x\right)$ are the normalized strain responses under the load l and m respectively, the corresponding CSD can be given as follows:

(2) $$CSD^{lm}=\int_{0}^{L_{x}}\frac{{\left(\varepsilon^{l}(x)-\varepsilon^{m}(x)\right)}^{2}}{\varepsilon^{l}(x)+\varepsilon^{m}(x)}dx$$

where $L_{x}$ represents the length of the wind turbine blade, and $CSD^{lm}$ denotes the CSD based on the strain response under the load *l* and *m.*

Suppose that the wind turbine blade is divided into N elements, the CSD of the *i*th element is defined as follows:

(3) $$CSD_{i}^{lm}=\int_{a_{i}}^{a_{i+1}}\frac{{\left(\varepsilon_{i}^{l}(x)-\varepsilon_{i}^{m}(x)\right)}^{2}}{\varepsilon_{i}^{l}(x)+\varepsilon_{i}^{m}(x)}dx$$

where $\left(a_{i},a_{i+1}\right)$ represents the coordinate of the *i*th element, $\varepsilon_{i}^{l}(x)$ and $\varepsilon_{i}^{m}(x)$ denote the normalized strain response of the *i*th element under the load *l* and *m* respectively, and the $CSD_{i}^{lm}$ represents the CSD of the *i*th element under the load *l* and *m*.

For the damage detection in the wind turbine blade, the CSD under different static loads are regarded as individual local decisions, so how to obtain a global and optimal decision based on them should be taken into account. Then, the feature information fusion method (FIF) is adopted in order to retain the advantage and abandon the shortcoming of the individual information sources at the same time, which is introduced in the next section.

### 2.2. The Feature Information Fusion (FIF)

In the FIF, judgments and decisions are made on the basis of the past information, and they are modified regularly when additional information become available. This is particularly important when a subjective decision has to be made, initially, with the lack of information, and then refined subsequently with later incoming information.

Suppose that there are two information sources $S_{1}$ and $S_{2}$, and NE objects need to be identified, which can be expressed as $A_{1},A_{2},A_{3},\cdots A_{NE}$, so the probability of each object is denoted as $P(A_{2}),P(A_{3}),\cdots ,P(A_{NE})$ respectively, let the kernel function and index function of the information source be $k\left(A_{i}\right)$ and $M\left(A_{i}\right)$, respectively, so the probability of each object can be calculated as follows:

(4) $$P\left(A_{i}\right)=M\left(A_{i}\right)\cdot k\left(A_{i}\right)\left(i=1,2,3,...,\text{NE}\right)$$

then the conditional probability of each object has been obtained; that is, $P\left(S_{1},S_{2}|A_{i}\right)$ is already known, so the conditional probability $P\left(A_{i}|S_{1},S_{2}\right)$ of the object $A_{i}$ can be given as follows:

(5) $$P\left(A_{i}|S_{1},S_{2}\right)=\frac{P\left(S_{1},S_{2}|A_{i}\right)P\left(A_{i}\right)}{\sum_{j=1}^{\mathrm{NE}}P\left(S_{1},S_{2}|A_{j}\right)P\left(A_{j}\right)}$$

When the decision of each information source is considered as independent, the above equation can also be written as:

(6) $$P\left(A_{i}|S_{1},S_{2}\right)=\frac{P\left(S_{1}|A_{i}\right)P\left(S_{2}|A_{i}\right)P\left(A_{i}\right)}{\sum_{j=1}^{\mathrm{NE}}P\left(S_{1}|A_{j}\right)P\left(S_{2}|A_{j}\right)P\left(A_{j}\right)}$$

It is crucial to select the information on the damage location as the feature information, and the maximal probability of each object is considered as the feature information in the FIF, so Equation (6) can be written as:

(7) $$P\left(A_{i}|S_{1},S_{2}\right)=\frac{\max\left(P\left(S_{1}|A_{i}\right),\;P\left(S_{2}|A_{i}\right)\right)P\left(A_{i}\right)}{\sum_{j=1}^{NE}P\left(S_{1}|A_{j}\right)P\left(S_{2}|A_{j}\right)P\left(A_{j}\right)}$$

If there is M independent information sources need to decided, the above procedure of selecting the feature information should be done twice. Let the maximum of the vector

$$\left\{P\left(S_{1}|A_{i}\right),\;P\left(S_{2}|A_{i}\right),\;\cdots \;P\left(S_{\text{M}}|A_{i}\right)\right\}$$

be the feature information denoted as $\max_{1}(P\left(S_{k}|A_{i}\right))$, and then the maximum of the above is assumed to be zero, so the vector becomes

$$\left\{P\left(S_{1}|A_{i}\right),\;P\left(S_{2}|A_{i}\right),\;\cdots ,\;0,\;\cdots \;P\left(S_{\text{M}}|A_{i}\right)\right\}$$

To obtain the next feature information, the maximum of the above-modified vector is denoted as $\max_{2}(P\left(S_{k}|A_{i}\right))$ is obtained; therefore, the corresponding information fusion can be given as follows:

(8) $$P\left(A_{i}|S_{1},S_{2}\right)==\frac{\max_{1}(P\left(S_{k}|A_{i}\right))\cdot \max_{2}(P\left(S_{k}|A_{i}\right))}{\sum_{j=1}^{\mathrm{NE}}\prod_{k=1}^{M}P\left(S_{k}|A_{j}\right)P\left(A_{j}\right)}$$

In the process of obtaining the global and optimal decision, the triangle kernel is adopted as the kernel function of the information source, the CSD under the smallest static load is regarded as the index function in the information fusion, and corresponding ones under other loads are adopted as individual information sources. To retain the advantage and abandon the shortcoming of the CSD at the same time, the most important information of the above individual information sources is selected as the feature information, so the global and optimal decision is made for the damage detection.

Generally speaking, the main advantage of the proposed method is that the proposed FIF can fuse the information of the individual information for the damage detection. Secondly it doesn’t need the information of the intact wind turbine blade, so it is a non-baseline damage detection method, Finally, only the strain response in the windward side of the wind turbine blade is used, which is more consistent with the situation in service.

## 3. Experimental Validation

### 3.1. Experimental Setup

**Figure 1:**
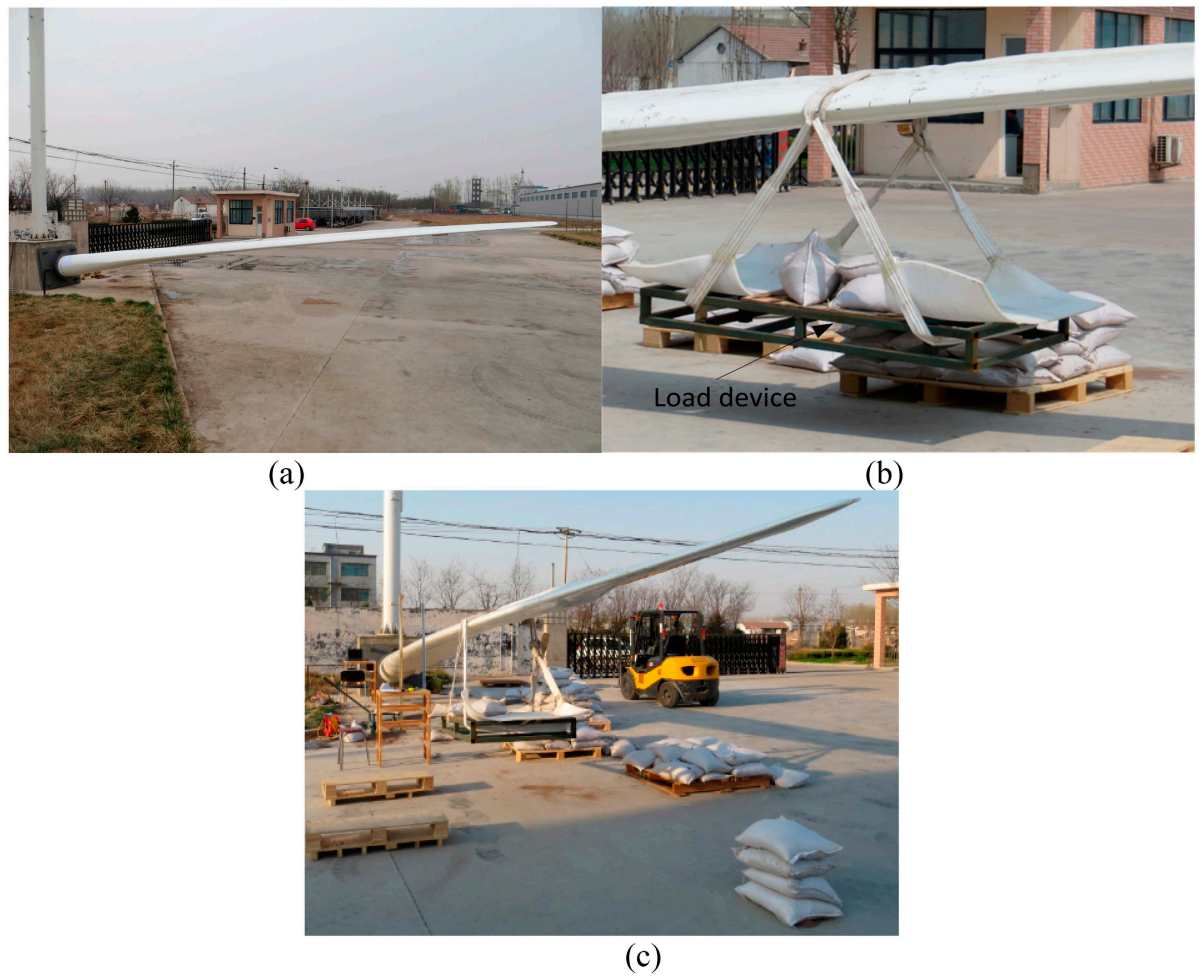
Experimental setup of the wind turbine blade. (**a**) Clamped wind turbine blade; (**b**) load device; and (**c**) global experimental setup.

A 220 kw, 13.2 m wind turbine blade is employed to describe the feasibility of the proposed method. The clamped blade is fixed by a concrete support structure shown in Figure 1a, and the distributed strain sensor system based on twelve FBG sensors is adopted to measure the strain response under the static load, which are adhered to the windward side of the wind turbine blade. The above FBG consists of the optical fiber and optical grating and there is optical fiber with the length of 5 m in the ends of the strain sensor system. Furthermore, the wavelength tolerance of the FBG is ±0.5 nm, the type of it is the single-mode SMF-28C FBG, and the length of the grating is 10 mm. The device shown in Figure 1b provides the method of imposing the load and the optical sensing interrogator SM130 and PC are employed as the demodulating and recording device. Since the FBG employed in this study is naked, a FBG protecting device is employed to give a steady room in order to protect the FBG in the process of imposing the load; the damage with dimensions of 8 × 1 × 0.5 cm is simulated by the electric saw in the surface of the blade. Finally, the global experimental setup is given in Figure 1c.

**Figure 2:**
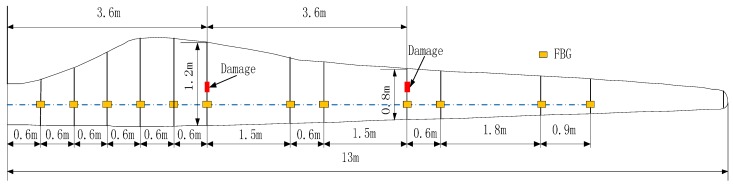
The schematic of the FBG and damage.

Figure 2 presents the location of each FBG and damage. The strain sensor system based on the FBG consists of twelve FBGs and the distance between adjacent FBGs is different. The dimension of the damage is 8 × 1 × 0.5 cm, the distance between measurement points and the damage is 4 cm, and the corresponding chords are 0.8 m and 1.2 m respectively, so the ratio of the damage length and the corresponding chord length is 0.1 and 0.0667 respectively.

### 3.2. Results and Discussions

**Figure 3:**
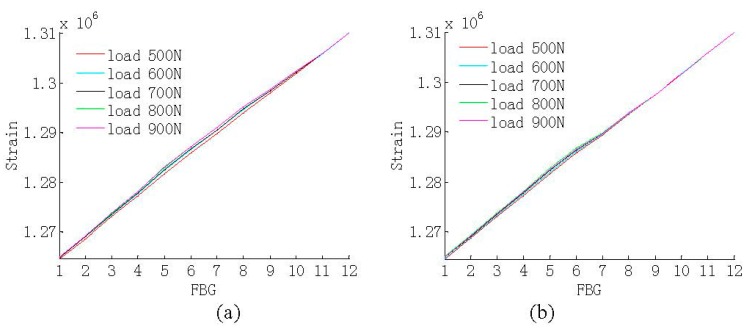
The strain distribution of the damaged wind turbine blade. (**a**) The single damage; and (**b**) two damaged regions.

In the above experiment, the static load is imposed by the same weight sandbags on one loading position, the distance between it and the root of wind turbine blade is 6.8 m. Then, the strain response under different magnitudes of the load is measured, and the range of the load is increased from 500 N to 900 N, in increments of 100 N, so the strain distribution of the above blade under five load magnitudes shown in Figure 3 is measured based on the distributed strain sensor system.

Suppose the strain response under the static load l on the *i*th FBG is denoted as ${\bar{\varepsilon }}^{l}\left(x_{i}\right)$, so the normalized strain response $\varepsilon^{l}\left(x\right)$ can be obtained as follows:

(9) $$\varepsilon^{l}\left(x_{i}\right)=\frac{{\bar{\varepsilon }}^{l}\left(x_{i}\right)}{\sum_{i=1}^{12}{\bar{\varepsilon }}^{l}\left(x_{i}\right)}$$

where *i* denotes the number of the FBG.

Therefore the normalized strain distributions can be regarded as different probability distributions, and it can be observed that the strain gets higher when the loads become greater. In addition, the self-weight of the wind turbine blade is ignored since the associated strains can be regarded as pre-existing before the application of the strain sensor FBGs.

The CSD is calculated between the above strain distributions; for example, it can be calculated between the strain distributions under the load of 500 N and 600 N respectively, then ten kinds of CSD are obtained, and the mean damage detection error of them is obtained, respectively, which will be introduced in Section 3.4, so the CSDs with first four minimal mean damage detection errors are used as the individual information for the damage detection.

**Figure 4:**
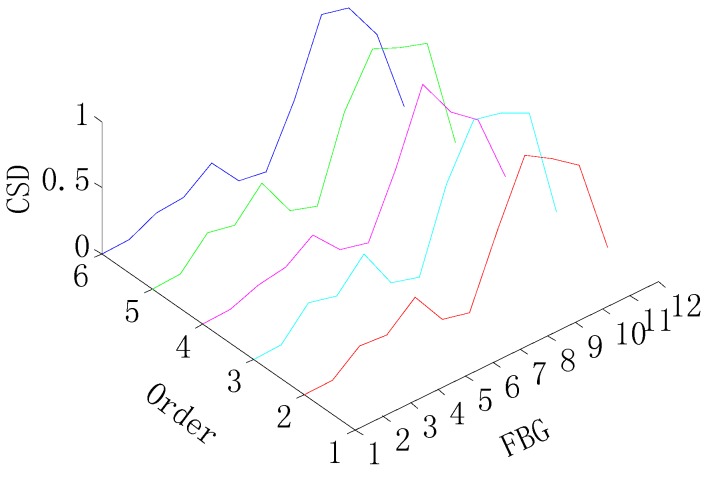
CSD for the single-damage detection.

**Figure 5:**
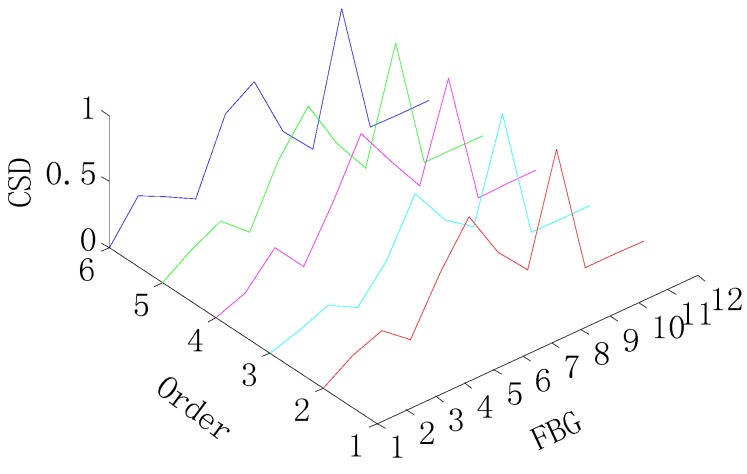
CSD for two-damage detection.

**Figure 6:**
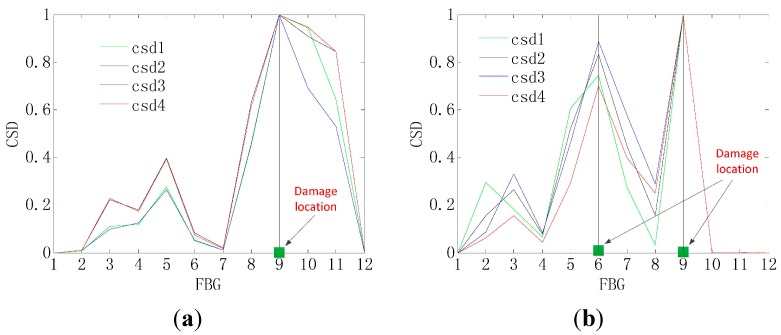
Method of detecting the damage location by the CSD. (**a**) The single-damage detection; (**b**) Two-damage detection.

The graphs shown in Figure 4 and Figure 5 provide the damage detection result by the CSD, and it can be shown that the shape change in the graph indicates the presence of the damage. Then, the damage location is obtained by the location of the shape change in the *x*-axis, as shown in Figure 6. Therefore, the CSD can detect the single and multiple (two) damage successfully, so the CSD can be regarded as an effective damage-sensitive feature, but a relative lower peak appears at a spurious damage location in the damage detection, which indicates the presence of damage where no damage, in fact, exists. Therefore, the information fusion method is needed to filter out this spurious damage location in the single damage detection.

In the FIF, the CSD under different static loads are regarded as individual information sources, and sources are independent among each other, so the CSDFIF based on the CSD is calculated.

**Figure 7:**
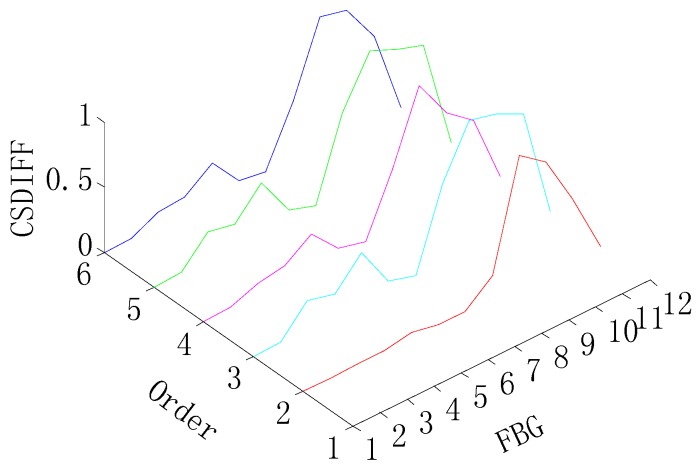
CSDFIF for the single-damage detection.

**Figure 8:**
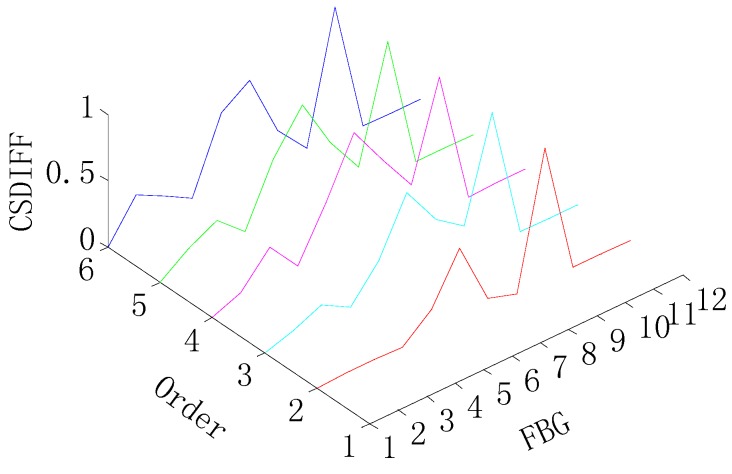
CSDFIF for two-damage detection.

**Figure 9:**
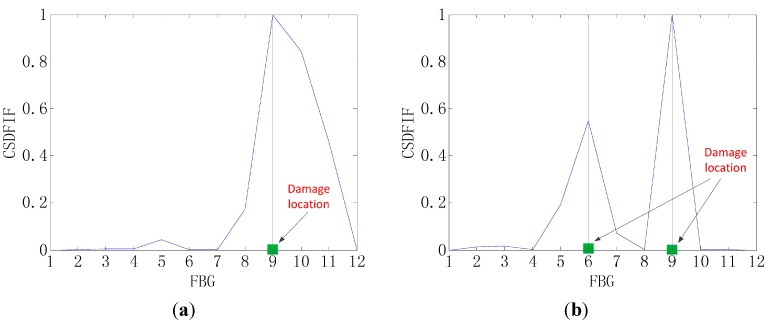
Method of detecting the damage location by the CSDFIF. (**a**) The single-damage detection; (**b**) Two-damage detection.

Figure 7 and Figure 8 give damage detection results by the CSDFIF for the single and multiple (two) damages and the damage location can also be obtained by the location of the shape change in the x-axis, as shown in Figure 9. For the single damage detection, there is no relative lower peak in the graph, so it can be observed that the information fusion method FIF has the ability of filtering out the spurious damage location, which is important in practice. For two-damage detection, the above conclusion can also be drawn, but the CSDFIF between two peaks on the damage location is relatively smaller, as compared with the CSD, which means a small error of the damage detection.

In addition, it is clear from Figure 7 and Figure 8 that the presence of damage produces a strain peak much more pronounced than that observed in Figure 4 and Figure 5, which means the greater accuracy of the damage detection. Therefore, the information fusion method, FIF, could be regarded as an effective and feasible approach for optimizing and showing information on the damage detection in the CSD.

Generally speaking, the proposed method can detect damage in the wind turbine blade accurately, and the CSD can be regarded as an effective damage-sensitive feature, the information fusion method FIF can optimize and show information on the damage detection in the CSD.

### 3.3. Comparison with the SEM

The strain energy method (SEM) is a classic method for the damage detection [16], which is employed to describe the advantage of the proposed method, and the strain energy based on the strain response under the static load *l* of the *i*th element in the wind turbine blade is given as follows:

(10) $$SEM_{i}=\frac{U_{i}^{l}}{U^{l}}=\frac{\int_{a_{i}}^{a_{i+1}}{\left(EI\right)}_{i}(\varepsilon_{i}^{l})dx}{\int_{0}^{L_{x}}\left(EI)(\varepsilon^{l}\right)dx}$$

where $SEM_{i}$ represents the strain energy of the *i*th element in the wind turbine blade.

**Figure 10:**
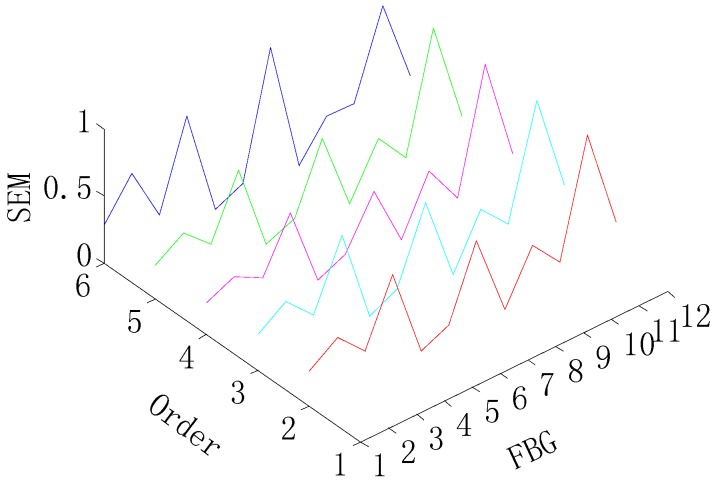
SEM for the single-damage detection.

**Figure 11:**
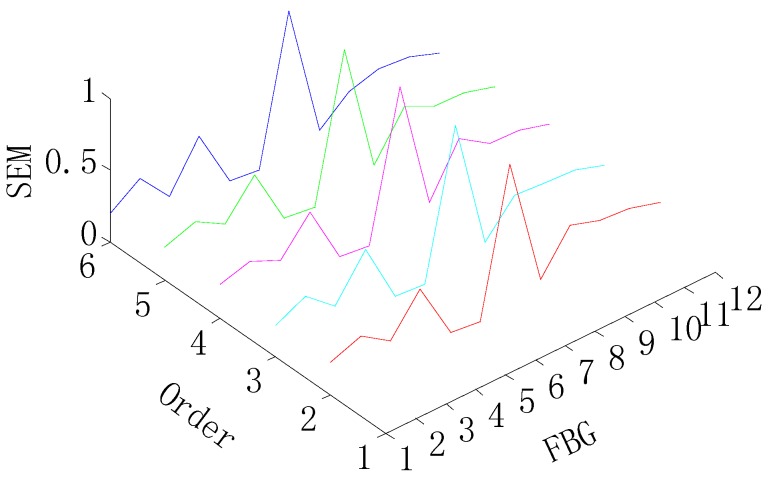
SEM for two-damage detection.

Figure 10 and Figure 11 present damage detection results by the SEM in order to describe the advantage of the proposed method, and it can be shown that the SEM is not able to detect the single damage, and can only detect damage on the FBG 6 in two-damage detection, as shown in Figure 11. The reason for this is there is no baseline available of the intact wind turbine blade in the SEM, so it can be said that the proposed method has significant advantage over the SEM.

### 3.4. Comparison of Damage Detection Error

**Figure 12:**
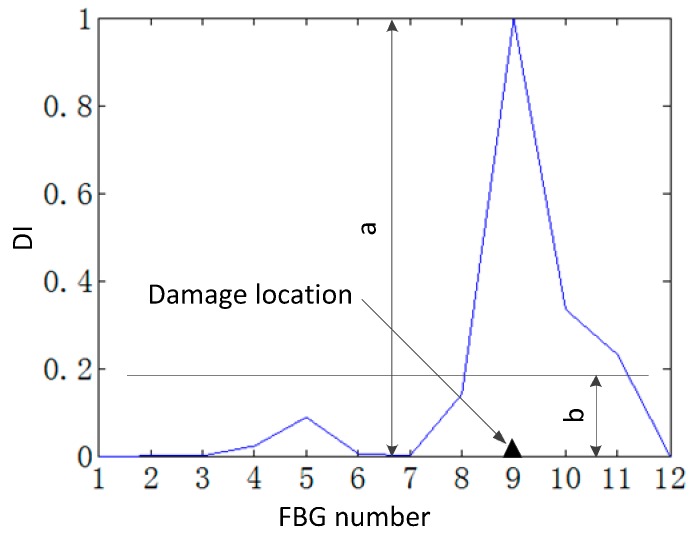
Mean damage detection error.

In order to evaluate the error of the proposed method, the error evaluation index MDDE is adopted. Let the maximal peak value of the damage index on the damage location and the mean of the damage index in the intact region be a and b, respectively, so the MDDE is defined as the ratio of a and b, as shown in Figure 12.

The result shown in the Table 1 provides the MDDE by the proposed method and the SEM, and it can be shown that the MDDE of the CSD is much larger than that of the CSDFIF, which is consistent with the above study.

**Table 1:** MDDE of the wind turbine blade.

DI	Single Damage	Two Damages
CSD	0.23863	0.12738
CSDFIF	0.12786	0.024546
SEM	0.30748	0.69678

The MDDE of the CSDFIF for two-damage detection is minimal, and the MDDE of the SEM is maximal. The MDDE of the SEM is larger than those of the CSDFIF and CSD, respectively. Therefore, it can be concluded that the proposed method shows a significant advantage over the SEM.

Generally speaking, the MDDE takes into account of the global damage detection error, and the proposed method can detect damage more accurately than the SEM by the comparison of the MDDE.

## 4. Conclusions

In this paper, a new damage detection method using the strain response under the static load is presented, and the following conclusions can be drawn:

(1) For the single and multiple (two) damage detection in the wind turbine blade, it can be observed that the proposed method can detect the presence of damage and give the damage location in a relatively higher accuracy than the SEM.
(2) The CSD can be regarded as an effective damage-sensitive feature and the individual information source for the damage detection in the wind turbine blade.
(3) The information fusion method, FIF, can fuse and optimize information in the damage-sensitive feature, CSD, for a global decision on the damage detection, and the proposed method CSDFIF shows an advantage over the SEM by the comparison the MDDE value.
(4) The proposed method CSDFIF is thought to be a very practical technique for structural health monitoring because it is robust and the minimal historical data that is needed.
